# Editorial: Mycorrhiza in Tropical and Neotropical Ecosystems

**DOI:** 10.3389/fpls.2018.00308

**Published:** 2018-03-22

**Authors:** Mohamed Hijri, Amadou Bâ

**Affiliations:** ^1^Département de Sciences Biologiques, Institut de Recherche en Biologie Végétale, Université de Montréal, Montréal, QC, Canada; ^2^Université des Antilles, LBPV/LSTM/UMR113, Guadeloupe, France

**Keywords:** ectomycorrhiza, arbuscular mycorrhiza, biofertilizer, diversity analysis, abiotic stresses

Mycorrhizal symbiosis is a mutualistic plant-fungus association that plays a major role in the function, maintenance and evolution of biodiversity and agroecosystems stability and productivity. The fungus provides mineral nutrients, water, protection against pathogens, alleviation of abiotic stresses such as salinity, drought and pollution, to the plant which, in return, provides carbon as an energy source to fungus. Threats to biodiversity destruction in tropical and neotropical agroecosystems should encourage fast inventory of the diversity and function of mycorrhizae in tropical latitudes which are biodiversity hotspots. Mycorrhizal fungi and Plant Growth Promoting Rhizobacteria (PGPR) are important components in forestry and agriculture which have encouraged their utilization over the past decades (Diédhiou et al., 2005; Hijri, 2016). Mycorrhizal- and PGPR-based commercial inoculants are sold world-wide as biofertilizers in a variety of formulations in agriculture, horticulture and even in forestry. However, the success of these products is variable because of limited data on large-scale field applications particularly in tropical and sub-tropical agrosystems (Hijri, 2016).

Ectomycorrhizal (EM) associations can influence plant community assembly and facilitate plant coexistence in boreal and temperate regions (van der Heijden et al., 1998), but little is known in tropical and neotropical forests (Bâ et al., 2012; Ebenye et al., 2017). To this end, Essene et al. compared assembly patterns of EM fungi in bulk soil to EM root tips collected from three ecologically distinct species of dipterocarp in a Bornean lowland tropical rain forest. They found that soil type had a stronger role than Dipterocarp host species in shaping the EM fungal community.

Asmelash et al. presented a review on the potential role of arbuscular mycorrhizal fungi (AMF) to significantly improve successful restoration success of degraded lands where levels of infective AM abundance and diversity are often low. They concluded that successful restoration of infective propagules can potentially improve the restoration success of degraded lands.

Symanczik et al. compared the performance of three non-native AMF strains (*Rhizoglomus irregulare, Claroideoglomus claroideum*, and *Cetraspora helvetica*) with native communities contained in three soils (conventional, organic, and permaculture) from naranjilla plantations in Ecuador. The growth response experiment has shown that two of the three non-native AMF, a mixture of the three and soil from a permaculture site led to significantly better acquisition of phosphorus (up to 104%) compared to non-inoculated controls. These results suggest that the use of non-native AMF and local soils as inoculants represent a valid approach to improve nutrient uptake efficiency of naranjilla. In another study, Cely et al. compared the effect *Rhizophagus clarus* (syn. *Rhizoglomus clarus*) and a supply of conventional fertilizer on growth and yield of two crops, soybean (*Glycine max* L.) and cotton (*Gossypium hirsutum* L.), under field conditions in Brazil. They showed that mycorrhizal inoculation significantly increased root colonization (~20%), P and N content as well as crop yield in both inoculated soybean and cotton. The authors conclude that *R. clarus* inoculation increased the effectiveness of fertilizer application in soybean and reduce the fertilizer dosage in cotton. Séry et al. used different approaches based on the selection of native AMF (*Acaulospora colombiana* and *Ambispora appendicular*) and their application in cassava crop production in greenhouse trials and under field conditions in Ivory Coast. Greenhouse trials showed that *A. colombiana* significantly improved the growth of cassava and enhanced tolerance to water stress. Furthermore, combined inoculation of *A. colombiana* and *A. appendicula* enhanced plant resistance against nematode attacks. In field conditions, the *A. colombiana* single inoculation and the dual inoculation significantly improved cassava yield compared to the control. However, no significant difference was observed between native and commercial inoculants which shows that interactions of introduced mycorrhizal inoculants and native communities can provide contrasting results.

The fundamental importance of mycorrhizal associations in tropical agrosystems is not restricted to crops, but extends to forestry as Cely et al. tested two native AMF *Claroideoglomus etunicatum* and *Acaulospora* sp., two native strains of *Rhizobium* sp., and one non-native PGPR strain of *Burkholderia* sp. on wood production of a fast-growing tree *Schizolobium parahyba* var. *amazonicum* (Huber ex Ducke) in Brazil under field conditions. Different combinations of microbial inoculants were complemented with two doses of conventional fertilizers in two planting methods, seed sowing and seedling planting. Among all combinations, two of them have shown a significant increase of wood yield or seedling growth in each planting method. The authors concluded that inoculation of *S. parahyba* with AMF and PGPR increased wood yield by approximately 20% compared to the application of fertilizer alone.

Arbuscular mycorrhizal fungi have been largely used for alleviating stress effects on host-plants by increasing the nutrient availability and enhancing the productivity in these plants (Dodd and Pérez-Alfocea, 2012). In extreme polluted environments by petroleum hydrocarbon and trace elements (de la Providencia et al., 2015), we do not yet have any clear evidence that AMF directly degrade petroleum hydrocarbon, however, they might stimulate soil metabolic activity of microorganisms, particularly bacteria and fungi, resulting to an acceleration of the immobilization and translocation of trace elements and the degradation of organic pollutants.

Nath et al. presented a mini review on reactive oxygen species (ROS) balance between its generation and scavenging, which is an essential indicator of adaptive defense response of plants under biotic and abiotic stresses. They showed that AMF and *Piriformospora indica* are well-known to colonize plant root, to enhance ROS-metabolism and to maintain ROS-homeostasis.

Chandrasekaran et al. conducted a comprehensive meta-analysis on AM efficiency on C3 and C4 plants under salt stress across 60 published studies. The authors compared the response of some parameters such as AMF and plants identities, soil textures, experiment conditions (greenhouse *versus* field), and the results clearly showed a positive effect on plants upon AM inoculation under salinity stress, regardless of the photosynthetic pathway. However, the authors found that C3 plants showed significantly more mycorrhizal inoculation than C4 plants. Interestingly, the meta-analysis showed that single inoculation with *R. irregularis* had a positive effect on C3 plants while inoculation with *Funneliformis mosseae* had a positive effect on C4 plants.

Using a greenhouse trial, Huang et al. tested the effect of the AMF *F. mosseae* on the subcellular compartmentalization and chemical forms of lead (Pb) in black locust (*Robinia pseudoacacia* L.), a plant species that is tolerant to Pb in polluted soils. Inoculation with *F. mosseae* significantly increased the proportion of Pb in the cell wall and soluble fractions, while it decreased the proportion in the organelle fraction of roots, stems, and leaves. Interestingly, AM inoculation increased the proportion of inactive Pb and reduced the proportion of water-soluble Pb in the roots, stems, and leaves.

## Conclusion

Overall, the studies presented in the issue of “Mycorrhiza in Tropical and Neotropical Ecosystems” have documented the effectiveness of commercial and native mycorrhizal inoculants in tropical agrosystems and forestry. The results demonstrate that inoculation with either native or non-native AMF inoculants improved crop yield under field conditions.

## Author contributions

All authors listed have made a substantial, direct and intellectual contribution to the work, and approved it for publication.

### Conflict of interest statement

The authors declare that the research was conducted in the absence of any commercial or financial relationships that could be construed as a potential conflict of interest.
